# PATHOLOGICAL TAU ABORTS CELL CYCLE ACTIVATION BY MIMICKING EPITHELIAL-MESENCHYMAL-TRANSITION IN NEURONS

**DOI:** 10.1093/geroni/igz038.3075

**Published:** 2019-11-08

**Authors:** Roseann V Phan, Adrian Beckmann, Bess E Frost

**Affiliations:** 1 Howard University, Washington, District of Columbia, United States; 2 University of Texas San Antonio, San Antonio, Texas, United States; 3 University of Texas Health San Antonio, San Antonio, Texas, United States

## Abstract

Alzheimer’s disease (AD) is an irreversible neurodegenerative disorder which is characterized by neurofibrillary tau tangles and amyloid-ß plaques. Our laboratory uses tau transgenic Drosophila to rapidly test hypotheses along with human brain samples in order to ensure that our work is relevant to clinical endeavors. Using this approach, we identified a neurodegenerative pathway whereby pathological tau over-stabilizes filamentous actin (f-actin), leading to disrupting aberrant nuclear pleomorphisms and decondensation of heterochromatic DNA. Due to the neuronal phenotypes observed in tau-transgenic Drosophila and reentry of post-mitotic neurons into the cell cycle, we hypothesized that tau perturbs the cellular program that maintains terminal neuronal differentiation. Based on RNA-sequencing, we identified prospero as the most differentially expressed and downregulated transcript in tau transgenic flies. Prospero regulates genes that promote and maintain terminal neuronal differentiation. At the protein level, prospero is significantly reduced in tau transgenic flies at 10 days old. We find that over stabilization of f-actin or over-expression of moesin, a cytoskeletal protein known to participate in tumor progression and metastasis, depletes prospero. Using genetically manipulated prospero target genes we find a connection to neurodegeneration. These targets include a PKC, tep4, and knot-suggesting that broad cellular processes are affected by this reduction. Overall, our findings suggest that pathological tau causes cell cycle re-entry by disrupting transcription factors governing terminal neuronal differentiation as well as over-stabilization of f-actin being the driving loss of terminal neuronal differentiation and casually associated with neuronal death.

